# Adaptive Approach to Treating Cervical Cancer in a Patient With Dramatic Uterine Movement

**DOI:** 10.7759/cureus.72938

**Published:** 2024-11-03

**Authors:** Mohammad Ghafouri, Steven Miller, Jay Burmeister, Ramesh Boggula

**Affiliations:** 1 Department of Oncology, Wayne State University School of Medicine, Detroit, USA

**Keywords:** adaptive radiation therapy, high-dose rate (hdr) brachytherapy, radiation, radiation and clinical oncology, uterine cervical cancer

## Abstract

Adaptive radiation therapy is a modern technological advancement that allows radiation treatments to be adjusted daily to account for changes in the patient's anatomy, such as bladder and rectal filling, as well as changes in the tumor volume and position. In this case report, we present a patient with locally advanced cervical cancer who received definitive radiation therapy of 4500 cGy in 25 fractions using the Varian's Ethos system. We observed substantial daily uterine movement, which required re-optimization of each treatment fraction. Without the daily plan adaptation, the treatment would have resulted in markedly suboptimal dose coverage to the tumor. This case report highlights the importance of adaptive radiotherapy in managing anatomical changes in cervical cancer treatment and improving outcomes.

## Introduction

Cervical cancer is a prevalent malignancy affecting women worldwide. It is highly associated with high-risk human papillomavirus (HPV) types, notably HPV-16 and HPV-18 infections [1,2]. However, despite widespread screening and HPV vaccination, it remains a significant health burden globally. According to the American Cancer Society, the estimated number of new invasive cervical cancer cases in the United States for 2024 is 13,820, with an estimated 4,360 deaths [3,4].

Locally advanced cervical cancer (LACC) refers to cervical cancer that has spread beyond the cervix but has not yet metastasized to distant organs. It typically includes stages IIB to IVA of the FIGO (International Federation of Gynecology and Obstetrics) staging system [5]. The standard treatment for LACC typically involves chemoradiotherapy, combining external beam radiation therapy (EBRT) to the pelvis (and possibly extended-field radiation if para-aortic lymph nodes are involved) with concurrent chemotherapy, usually cisplatin, to enhance radiation effectiveness [6-9] and immunotherapy. Brachytherapy (internal radiation) often follows EBRT. Surgery is not usually a first line of treatment but may be considered post-chemoradiotherapy for residual tumors or palliative purposes. In recurrent or metastatic cases, targeted therapies or immunotherapies, such as pembrolizumab, may be considered, particularly if the tumor has high PD-L1 expression [10].

Adaptive radiation therapy (ART) is a modern radiotherapy technique that adapts treatment plans based on near real-time anatomical changes observed immediately before treatment. Managing LACC is particularly challenging due to substantial inter-fraction anatomical changes in the uterus and cervix. These changes are often due to variations in rectal and bladder filling and potential tumor shrinkage over the course of radiotherapy [11-12]. ART addresses these issues by allowing daily adaptation of the radiation treatment plan based on daily cone beam computed tomography (CBCT). This dynamic plan adaptation improves targeted tissue coverage with the prescription radiation dose. It reduces side effects on the bowel and bladder by minimizing the dose to normal tissue structures [13-15].

In this case report, we present a patient who had locally advanced cervical cancer with emphasis on the importance of ART in managing the uterus and cervix.

## Case presentation

**Figure 1:**
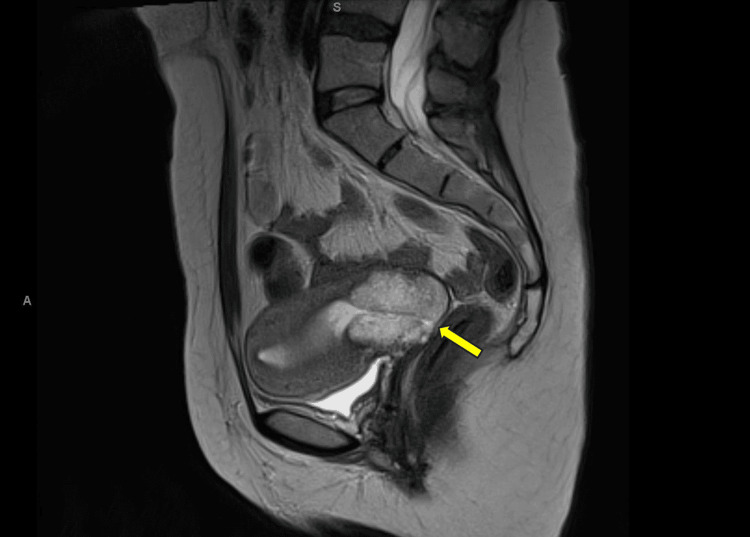
Sagittal T2 MRI. The yellow arrow is pointing to the cervical lesion.

The patient is a female in her 40s with a history of cervical dysplasia. She underwent a snare biopsy of the cervix in Lviv, Ukraine, and pathology was consistent with a moderate to poorly differentiated adenocarcinoma. She subsequently underwent additional staging studies, including an MRI of the pelvis, which revealed a 49 x 40 mm cervical mass with no pelvic lymphadenopathy detected (Figure 1).

**Figure 2:**
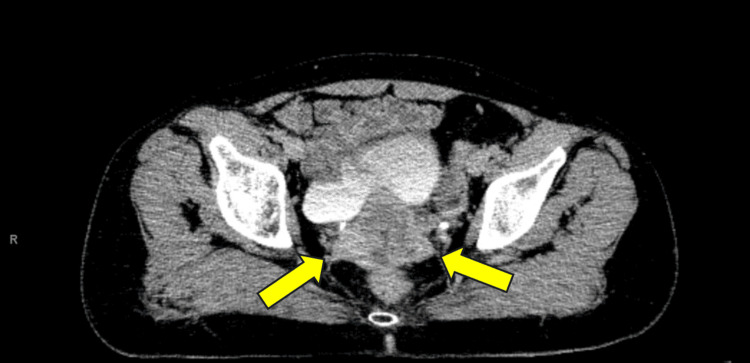
Axial CT image with the yellow arrows pointing to the cervical mass, which measured over 5 cm in size at presentation.

A CT scan of the abdomen and pelvis demonstrated a 50 x 42 mm cervical tumor (Figure 2).

She received four cycles of cisplatin and taxol in Ukraine, which she tolerated well, and then transferred her care to the United States.

She underwent a re-evaluation, including an examination under anesthesia, which revealed a cervix measuring 4.5 cm, symmetrically indurated, without any visible or distinct masses. Cervical biopsies were obtained, and pathology was consistent with scant residual neoplastic epithelium in a background of extensive mucinous material, consistent with the treatment effect. The neoplastic epithelium had mucinous features, including focal goblet cells and rare angulated neoplastic glands in fibromuscular tissue, consistent with focal residual invasive adenocarcinoma. 

Additional staging studies, including a positron emission tomography scan (PET) at an outside institution, revealed no PET evidence of disease. Her case was discussed in the gynecology oncology multidisciplinary tumor board. Since the patient had not received the standard of care therapy for cervical cancer, she was recommended to undergo a course of external beam radiation therapy as well as intracavitary brachytherapy with weekly cisplatinum and immunotherapy.

An overview of the adaptive radiation therapy workflow

The Ethos treatment delivery system (Varian Medical Systems, Palo Alto, CA) is an advanced technology platform that integrates ART into daily clinical practice. It includes iterative CBCT imaging, AI-based automated contouring of influencer structures, treatment plan optimization, plan evaluation, and treatment delivery. Starting with a conventional RT workflow, it diverges once daily CBCT imaging is acquired to facilitate the adaptation of the radiotherapy plan to anatomical changes of both targets and critical structures. Ethos provides automated contouring followed by manual contour review and optimization of the adaptive plan. The system then generates two plans: a "scheduled" plan that recalculates the daily dose using the initial treatment plan applied to the current CBCT and an "adaptive" plan that reoptimizes the treatment plan to account for changes in the shapes and locations of targets and normal tissue structures. These plans are then compared, and the user selects the preferred plan for treatment for that session [16-17].

Patient treatment planning

The patient underwent a course of primary radiation and chemotherapy to the pelvis and para-aortic lymph nodes. The gross tumor volume (GTV) consisted of the cervix. The clinical target volume (CTV) included the cervix with a 10 mm expansion plus the uterus, proximal vagina, and para-aortic lymph nodes. The planning target volume (PTV) included an additional 5 mm expansion around the CTV. At the time of simulation, a CT scan with a full bladder was obtained. A smaller PTV margin was used rather than a more conventional 10-15 mm expansion around the uterus since the patient was treated with ART on the Ethos system. The prescribed dose was 4500 centigray (cGy) delivered in 25 fractions (180 cGy per fraction). Table 1 provides a detailed overview of the clinical goals and the corresponding results in the original treatment plan.

**Table 1:** Comparison of the target and organ-at-risk objectives as defined during the Ethos treatment planning process with the results achieved in the original treatment plan. PTV_4500= planning target volume receiving 4500 cGy. CTV = clinical target volume, CTV_LN = clinical target volume including lymph nodes, Dmax = maximum dose, Vx% = volume receiving x% of the dose

Structures	Goals	Original Plan
			PTV_4500	V98% ≥ 98%	98.4%
	V100% ≥ 95%	96.5%
	Dmax(0.03 cm3) ≤ 108%	106.1%
CTV	V95% ≥ 95%	100.0%
CTV_LN	V95% ≥ 95%	100.0%
Bladder	Dmax ≤ 4725 cGy	4724 cGy
Femur head and neck left	Dmax ≤ 4725 cGy	3308 cGy
Femur head and neck right	Dmax ≤ 4725 cGy	3090 cGy
Liver	Dmax ≤ 4725 cGy	1975 cGy
Kidney left	V1800 cGy ≤ 10%	5.4%
Kidney right	V1800 cGy ≤ 10%	5.0%
Rectum	Dmax ≤ 4725 cGy	4649 cGy
Spinal Canal	Dmax ≤ 4500 cGy	2223 cGy
Bowel-CTV	Dmax ≤ 4725 cGy	4663 cGy

These goals were used for generating the initial treatment plan and also for daily on-couch plan optimization. The initial plan was calculated using the CT acquired during treatment simulation (sim-CT) acquired on a Brilliance Big Bore CT scanner (Philips Medical Systems, Best, Netherlands). A two-isocenter plan was necessary for adequate coverage of the target (craniocaudal length greater than 260 mm), resulting in an 18-field IMRT plan (9 fields per isocenter). The total number of monitor units (MUs) for the entire initial plan was 2826.

Daily treatment adaptation

**Figure 3:**
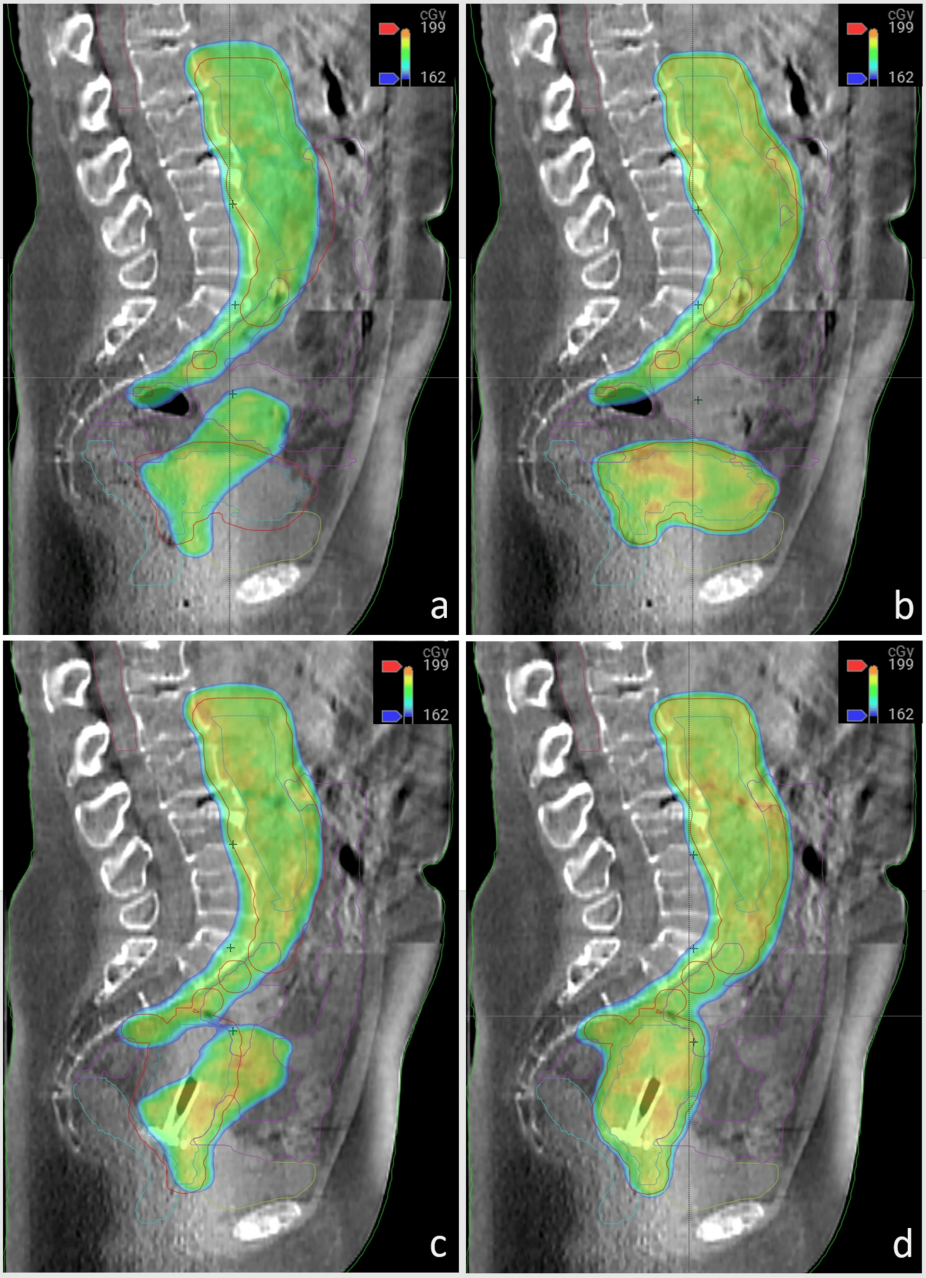
Two example treatment fractions demonstrating the substantial daily variation in the uterus and surrounding critical organs. Scheduled (not adapted) plans for these two treatment days are displayed in panels a) and c), while the associated adapted (re-optimized) plans are shown in panels b) and d). Use of the scheduled plan would have resulted in the suboptimal planning target volume coverage and a higher delivered dose to the bowel.

Significant variations in the shape and size of the uterus and surrounding structures were observed during the daily online adaptive process. Considerable daily variation in the uterus and critical organs for two treatment days (Figure 3).

These changes resulted in suboptimal target coverage by the prescription dose and higher doses to critical organs in the ‘scheduled’ plans (i.e., plans without adaptive re-optimization). Consequently, "adapted" treatment plans were necessary for all 25 treatment fractions to consistently achieve optimal target coverage and critical organ goals. The adapted plans successfully met the target goals and maintained critical organ tolerance for each fraction. 

Evaluation of DVH parameters

We compared the DVH parameters between the adapted and scheduled plans for various metrics: V95% for the CTV and CTV lymph nodes, V98%, V100%, and Dmax (0.03cc) for the PTV, and Dmax (maximum dose) for all critical organs including the bladder, bowel-CTV, femoral heads, kidneys, liver, rectum, and spinal canal. In addition, we compared the DVH parameters of the adapted and scheduled plans to the original plan.

## Discussion

In this study, we evaluated adapted and scheduled plans for cervical cancer treatment using the Ethos system. Our findings demonstrated that the adapted plans consistently outperformed the scheduled plans in terms of target coverage for every fraction. The dose-volume histogram (DVH) parameters for the original, adapted, and scheduled plans are summarized in Table 2.

**Table 2:** Comparison of clinical goals achieved between the scheduled and adaptive plans for the entire treatment course. The values shown for the adapted and scheduled plans are averages over the 25 treatment fractions. PTV_4500= planning target volume receiving 4500 cGy, CTV = clinical target volume, CTV_LN = clinical target volume including lymph nodes, Dmax = maximum dose, Vx% = volume receiving x% of the dose

Structures	Goals	Scheduled plans			Adapted plans		
		Average	SD	Range	Average	SD	Range
PTV_4500	V98% (%)	86.3	3.7	79.4-92.6	98.4	0.1	98.0-98.5
	V100% (%)	61.2	13.0	41.8-82.0	95.0	0.0	95.0-95.0
	Dmax(0.03 cm3) (%)	106.3	0.7	105.3-107.7	108.7	0.8	107.5-109.8
CTV	V95% (%)	66.0	19.8	37.2.0-100.0	100.0	0.0	100.0-100.0
CTV_LN	V95% (%)	100.0	0.0	99.9.0-100.0	100.0	0.0	100.0-100.0
Bladder	Dmax (cGy)	4636.0	71.5	4500.0-4750.0	4688.0	34.7	4650.0-4800.0
Femur head and neck left	Dmax (cGy)	2699.0	185.3	2275.0-3075.0	2901.0	397.5	2350.0-3400.0
Femur head and neck right	Dmax (cGy)	2683.0	202.8	2125.0-3025.0	2873.0	330.7	2350.0-3300.0
Liver	Dmax (cGy)	2762.0	333.2	1950.0-3250.0	2177.0	283.2	1975.0-2975.0
Kidney left	V1800 cGy (%)	1.9	0.4	1.3-3.2	4.2	1.8	2.4-7.5
Kidney right	V1800 cGy (%)	5.3	1.6	1.7-8.2	3.6	1.6	1.6-6.4
Rectum	Dmax (cGy)	4595.0	52.5	4450.0-4675.0	4670.0	25.0	4600.0-4725.0
Spinal canal	Dmax (cGy)	2142.0	61.1	1950.0-2225.0	2229.0	59.4	2175.0-2425.0
Bowel-CTV	Dmax (cGy)	4768.0	39.2	4700.0-4825.0	4812.0	41.5	4750.0-4925.0

**Figure 4:**
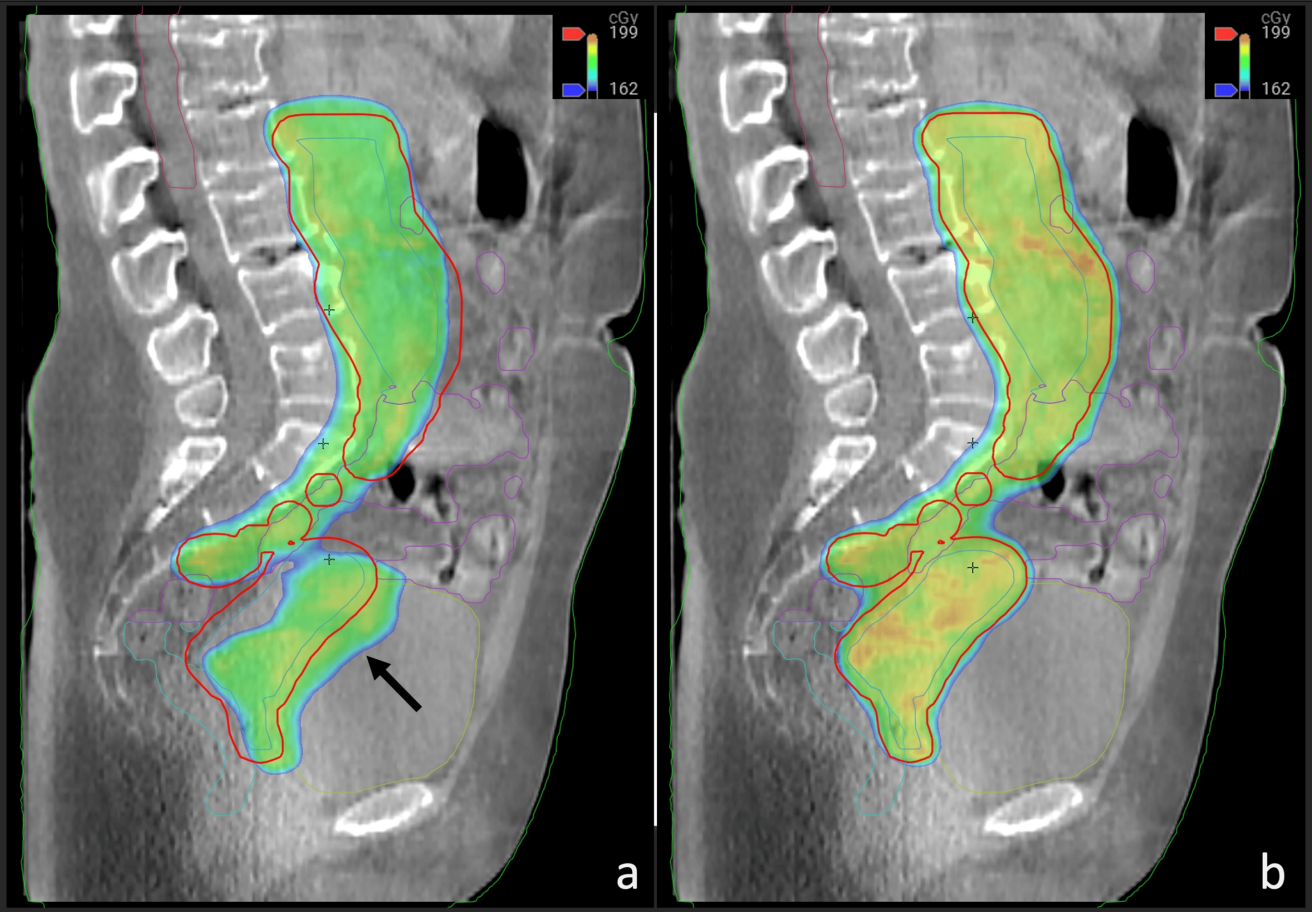
Example treatment fraction demonstrating the shift of the bladder into the high-dose region, as shown with a pointing arrow in panel a). Panel b) depicts the adapted dose distribution, which conforms to the target and avoids delivering high doses to the bladder.

Notably, the adapted plans provided superior coverage, with average CTV V95%, PTV V98%, and PTV V100% being 34%, 12%, and 34% higher, respectively, compared to the scheduled plans. In addition, the adapted plans resulted in a maximum dose (Dmax), on average, 21% lower for the liver than the scheduled plans. While differences in Dmax for other critical organs were relatively modest, substantial reductions in dose-volume characteristics were realized in the adaptive plans. For example, in fraction #5, the V90% for the bladder was reduced by 11% compared to the scheduled plan. The dose distribution between the scheduled and adapted treatment plans (Figure 4).

These findings align with the broader context of ART in gynecologic cancers, where CBCT-guided adaptive radiotherapy has proven to be a practical and effective option. Similar studies have highlighted the dosimetric benefits of ART with CBCT. For instance, a recent study evaluating 13 patients with cervical cancer (149 adaptive fractions) showed that the average time for delivering adaptive treatment was about 24 minutes, with a significant increase in V95% coverage by an average of 9.2%. In addition, there was a small but significant decrease in D2cc to the bladder, bowel, and rectum [17]. Another study reviewed 125 ART sessions using an AI-driven CBCT-guided system and found that the mean dose to PTV D98% was 96.7% and 94.9% in adapted and scheduled plans, respectively. The average treatment delivery time in these sessions was approximately 29 minutes. However, there was a decrease in D2cc to the bladder, bowel, and rectum, which was not significant [18]. In our study, the average treatment time for adaptive plans was approximately 33 minutes. In comparison, scheduled (non-adaptive) treatment plans have a shorter treatment time, typically by about two to five minutes. This difference is mainly because adaptive plans include an additional independent plan verification step, which is not required for scheduled plans.

To evaluate the clinical feasibility of adaptive ART using CBCT, Sibolt et al. treated five patients (three bladder and two lower gastrointestinal cancers) with an AI-driven and CBCT-based ART system. The high-dose PTV was significantly reduced by 42% due to margin reduction in bladder cancer cases [15]. In another study, 17 patients with endometrial and cervical cancers were treated with AI-driven CBCT-guided (iterative CBCT) ART. The dosimetric coverage of the vaginal PTV was significantly increased by nearly 7% (P < .05). The adapted plans showed that the mean bladder dose was about 105 cGy, and the mean rectum dose was 124 cGy. These values were 4 cGy lower than the mean doses in the scheduled plan [19].

**Figure 5:**
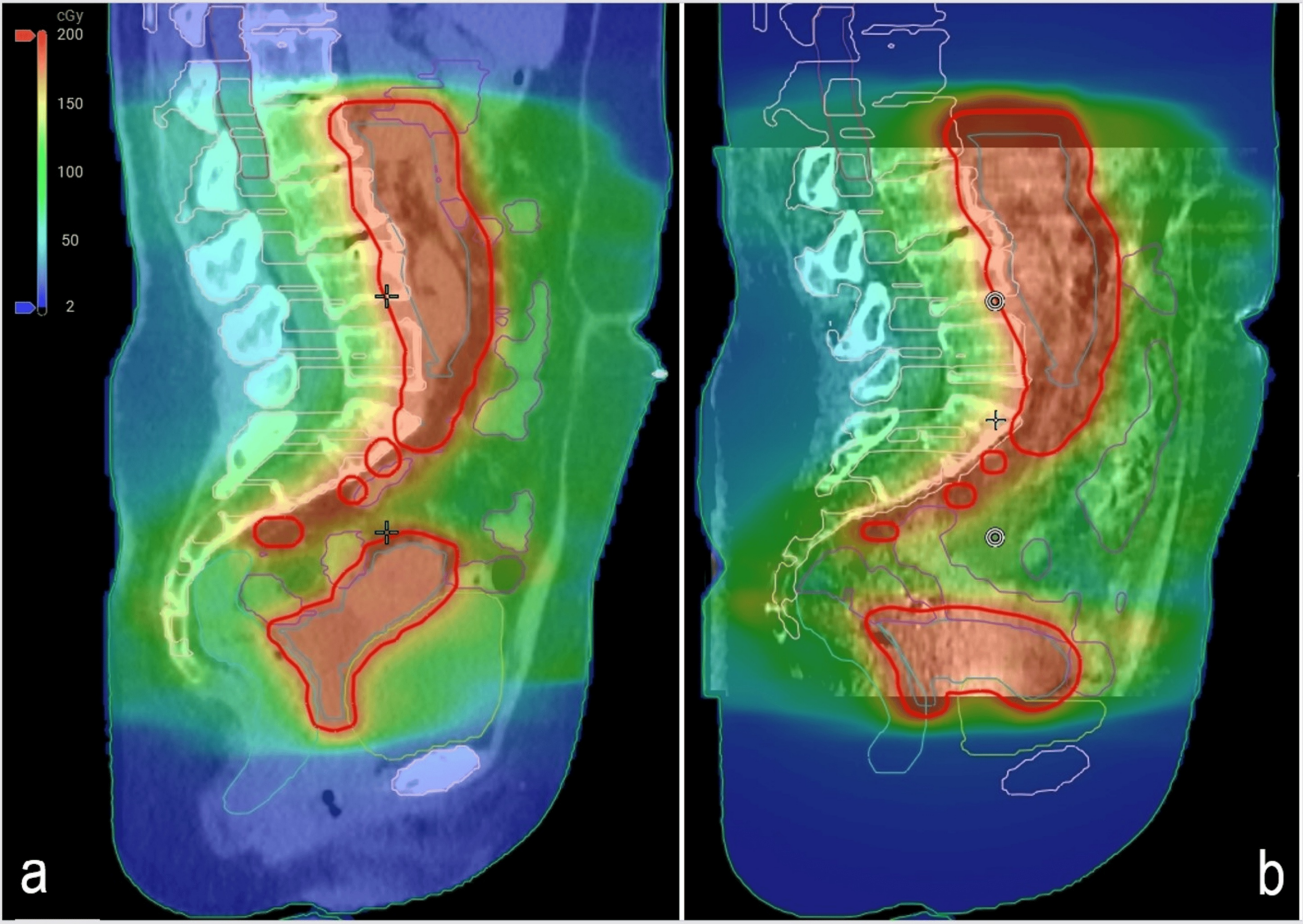
Comparison of planned versus reconstructed dose distributions for one of the treatment fractions.

Ethos reconstructs the 3D dose distribution based on the delivered MUs and the CBCT for each fraction. The planned versus delivered dose distribution for one of the treatment sessions (Figure 5).

Typically, two CBCTs are acquired per treatment session: the first CBCT is used for the adaptive workflow, and the second CBCT assesses intrafraction anatomical changes. In the future, contouring on the second CBCT could be used to evaluate the dose delivered to the patient. The availability of deformable image registration facilitates the summation of dose to individual volume elements within the patient, allowing the potential to evaluate the accumulation of dose to specific regions of target or normal tissue over multiple treatment fractions. Physicians can review the accumulated dose, perhaps every fifth fraction, and provide guidance for the remainder of the treatment.

## Conclusions

This case report highlights the importance of ART in treating locally advanced cervical cancer, particularly in cases with dramatic uterine movement. Using the Varian’s Ethos system, daily re-optimization of treatment plans not only improved tumor dose coverage but also minimized dose to critical organs. Without ART, such daily anatomical variability would likely result in suboptimal dose delivery, potentially impacting treatment effectiveness.

Our comparison of scheduled versus adapted treatment plans demonstrated that the adapted plans consistently achieved superior dosimetric plan quality. This was particularly evident in the superior target coverage achieved for every fraction throughout the treatment course. These findings advocate for incorporating ART into routine clinical practice, especially for patients with significant anatomical variability. Future studies should evaluate the dose accumulation over multiple fractions and correlate it with clinical outcomes.
